# The impact of mobile phone use on where we look and how we walk when negotiating floor based obstacles

**DOI:** 10.1371/journal.pone.0179802

**Published:** 2017-06-30

**Authors:** Matthew A. Timmis, Herre Bijl, Kieran Turner, Itay Basevitch, Matthew J. D. Taylor, Kjell N. van Paridon

**Affiliations:** 1 Cambridge Centre for Sport and Exercise Sciences (CCSES), Department of Sport and Exercise Sciences, Anglia Ruskin University, Cambridge, United Kingdom; 2 Centre for Sports & Exercise Sciences, School of Biological Sciences, University of Essex, Colchester, United Kingdom; University of Florida, UNITED STATES

## Abstract

Pedestrians regularly engage with their mobile phone whilst walking. The current study investigated how mobile phone use affects where people look (visual search behaviour) and how they negotiate a floor based hazard placed along the walking path. Whilst wearing a mobile eye tracker and motion analysis sensors, participants walked up to and negotiated a surface height change whilst writing a text, reading a text, talking on the phone, or without a phone. Differences in gait and visual search behaviour were found when using a mobile phone compared to when not using a phone. Using a phone resulted in looking less frequently and for less time at the surface height change, which led to adaptations in gait by negotiating it in a manner consistent with adopting an increasingly cautious stepping strategy. When using a mobile phone, writing a text whilst walking resulted in the greatest adaptions in gait and visual search behaviour compared to reading a text and talking on a mobile phone. Findings indicate that mobile phone users were able to adapt their visual search behaviour and gait to incorporate mobile phone use in a safe manner when negotiating floor based obstacles.

## Introduction

The number of people who own a mobile (cell) phone has increased dramatically in the last 30 years. In 1985, approximately 340,000 people owned a mobile phone in the United States (US). In 2010, this had risen to 302.9 million [1]. Recent surveys suggest that over 85% of people in the US and ~77% of the world’s population now own a mobile phone [2–4].

People engage with their mobile phone in a variety of ways, for example, making a telephone call, reading and sending text messages and emails, and engaging in online activities such as social networking and watching videos [5]. This increase in functionality resulted in 2.1 trillion manualized text messages sent, 2.2 trillion phone minutes used, [1] and 897 million people sending emails from their phone, something which is expected to rise to ~1.78 billion in 2017 [6].

Pedestrians regularly engage with their phone in a variety of ways whilst on the move. Approximately 62% of Americans report that they use their phone whilst ‘on the go’ [7]. Data on serious injuries involving road traffic and mobile phone use is sparse. Analysis of the National Electronic Injury Surveillance System (NEISS) for emergency departments between 2000–2011 identified 5,754 cases of emergency department admissions related to mobile phone use. Of these admissions, 310 were mobile phone related injuries [8] and 78% of the mobile phone related injuries were the result of falls [8]. In 2010, over 1,500 pedestrians visited hospitals in the US due to tripping, falling or walking into something whilst using their mobile phone. This was a marked increase from the number in 2007 (597 reported visits) and almost four times that of 2006 [9]; these figures are likely underestimated due to people attending the hospital failing to report phone use as the cause of the accident or not requiring hospital treatment for minor injuries [9].

The increase in pedestrian-phone related accidents has subsequently led to greater law enforcement for pedestrians crossing roads illegally when texting in several US towns and states [10] and in China, segregating footpaths for people walking and using their mobile phone and those walking without using a mobile phone [11].

The increase in pedestrian related accidents when walking and engaging with a mobile phone has led researchers to investigate the effect of phone use on pedestrian safety. Compared to walking without a mobile phone, adaptations in walking gait have been reported when texting, reading and talking on a mobile phone [3,12–16]. Phone use results in pedestrians walking slower, deviating more from a straight line or changing direction more, and demonstrating reduced situation awareness and/or inattentional blindness [3,12–16]. Importantly, the aforementioned gait research all required participants to walk along level terrain. In everyday life we frequently encounter complex terrain requiring adaptions in gait, for example negotiation of a change in surface height such as a step or kerb. There is currently little research investigating the impact of mobile phone use on adaptive gait.

The changes in walking gait that occur as a result of mobile phone use may be attributed to the altered visual search behaviour when engaging with the phone. Whilst walking and looking at the phone’s screen, pedestrians will not be able to acquire concurrent visual information from the fovea (central part of the eye which provides the highest level of visual acuity) of the surrounding environment to guide locomotion: something which has been previously highlighted as important in safe walking (e.g. [17,18]). When walking and texting or reading a text, the fovea is fixated on the phone’s screen. To acquire precise visual information from the environment, the eyes will need to re-fixate from the phone’s screen to an area within the environment. If this re-fixation does not occur frequently enough or long enough to sufficiently acquire an updated visual representation of the environment, an increased risk of accidents are expected as potential hazards will not be seen or seen without allowing enough time to plan/initiate a suitable response (i.e. walk round the hazard or step over the obstacle). Indeed, visual information is required in the previous step to successfully implement an adaptive strategy to safely step over or under an obstacle, and is required at least 2 steps in advance for changing direction (c.f. [19]). However, previous research has demonstrated that safe travel is still possible when a large proportion of time (~40%) is spent fixating at task irrelevant objects [20] or through relying on peripheral vision (aspect of vision which does not encompass the fovea) to step over an obstacle [21] or step up onto a surface height change [22] and when using a mobile phone, navigate whilst cycling [23] driving [24,25] and walking [14,16].

The current research addresses two important gaps in the literature;

1. Investigates whether adaptive gait is affected when required to walk up to and step onto a raised surface when engaging with a mobile phone (talking, texting or reading a text) compared to no phone being present. Much of the current literature has investigated how people navigate their environment through avoiding obstacles (i.e. route planning) whilst using their phone (e.g. [3,12–14,16,26,27]. Currently, there are few research studies investigating the effect of mobile phone use on obstacle negotiation. Negotiating an obstacle is a task that presents a high risk of injury since positioning of the foot in relation to the obstacle [28], the speed (velocity) and clearance height of the swinging limb when crossing the rising edge of the object [18] and foot placement on the upper level [29] all impact the risk of tripping / falling.
2. Investigates whether visual search behaviour changes dependent upon the manner in which the phone is being used. It is relevant to note that Oulasvirta et al. [30] highlighted that when using a mobile phone to search the internet, pedestrians looked at their screen in approximately four second bursts and made use of the time when the web browser was loading to acquire visual information from the surrounding environment. It is likely that when reading a text or email, rather than employ one long fixation, people will employ a series of shorter fixations to ‘glance’ at their phone to read whilst frequently fixating at the surrounding environment. However, with no previous research in this area, it is currently not known how adaptations in visual search strategy affect the pedestrian’s ability to safely negotiate floor based obstacles compared to no phone being present.

With the visual information acquired in advance of walking up to and negotiating a surface height change potentially impacting adaptive gait (i.e. negotiation over the floor based obstacle), we hypothesised that pedestrians will alter their visual search behaviour, which will subsequently impact their adaptive gait when using a phone compared to no phone being present. Furthermore, tasks which require the individual to fixate predominantly at the phone’s screen (i.e. typing a text and reading a text) will have a greater effect on visual search behaviour and adaptive gait.

## Method

### Participants

Twenty one participants (16 male, 5 female, age 25.4±6.2 years, height 178±14 cm, mass 78.0±15.4 kg, BMI 23.9±2.8 kg/m^2^; mean±SD) were recruited to the study. Participants were recruited via opportune sampling according to the following inclusion criteria. Participants, by self-report, were fit and healthy with no history of neurological or musculoskeletal disorders which could affect balance or gait and had either normal or corrected to normal vision (through wearing contact lenses) as determined through self-report: Participants were excluded if they wore spectacles as this interfered with eye tracking quality. All participants habitually used a touch screen smart phone, had used their current phone for a minimum of 6 months (range 0.5–3+ years) and all reported frequently using their mobile phone for texting, talking and reading a text whilst walking. Participants used their own phone in the study. The tenants of the Declaration of Helsinki were observed and Anglia Ruskin University’s Ethical Committee approved the study. Written consent was obtained from each participant prior to participation.

### Protocol

#### Experimental setup

**Fig 1:**
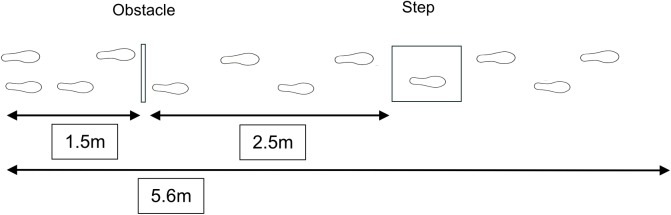
Schematic of the experimental set-up.

Participants walked along a 5.6m walkway, negotiating an obstacle and a surface height change. The obstacle, constructed from medium density fibreboard (0.5m width [medial-lateral dimension], 0.13m high [vertical dimension] and 0.012m deep [anterior-posterior dimension]), was positioned 1.5m from the start position. A step-up box (York Barbell, USA, 0.61m wide, 0.075m high and 0.61m deep) was used as the surface height change. The step-up box was positioned 3.95–4.05m from the start position and was constructed from plywood with a 1.27cm black rubber non-slip top. A black non-slip mat (width 1.5m length 15m) was laid on a light grey laboratory floor to indicate the walking path. Both the obstacle and surface height change were placed in the centre of the path (Fig 1).

The variation of +/- 10 cm in surface height change location was to ensure that participants did not become habituated with identifying and negotiating the surface height change located at the same distance from their start position.

A number of walking only trials (presented every third trial; a 1:3 ratio) were also completed. During these walking only trials, both the obstacle and surface height change were removed from the walkway, creating a level walkway. These trials were included to reduce the likelihood of participants becoming habituated.

#### Procedure

Prior to data collection, participants were instructed that for each trial, they were required to walk along the walking path where there may or may not be an obstacle present. Participants were told to safely step over the obstacle and avoid contacting / knocking over the hazard. Prior to the start of each trial, participants faced the opposite way from the intended walking direction. This particular start position was used to ensure participants did not receive advanced visual information from the environment prior to the start of the trial. Participants were told which of the following phone conditions they would complete immediately prior to the onset of the trial. Of note, for each phone condition, participants were not given any specific instruction regarding how to hold / interact with the phone. This was a purposeful strategy to ensure participants interacted with their phone in a manner reflective of being outside of the laboratory. The only specific instruction related to when talking on the phone (talk condition). Participants were instructed to keep the phone next to their ear and talk instead of using the speaker phone function. At the onset of the trial, the participant was free to select which foot to start (initiate gait) with (which varied from trial to trial). All participants completed the four different phone conditions three times, in a fully randomised order (i.e., 12 total trials);

1. No phone- Walking without a phone (phone was placed in the participant’s pocket).
2. Talking on their phone- at the start of the trial, participants were asked a question which they were required to answer whilst completing the gait task. Simple questions were asked which ranged in length from 23 to 42 (34±7, mean ± SD) characters, including spaces (e.g. ‘Have you seen any good films lately?’ or ‘What is your favourite type of music?’). During this condition, participants spoke into the phone to answer these questions.
3. Read a text message–prior to the start of the trial, the experimenter sent a text message to the participant’s phone. Participants began the trial immediately upon opening the text message. Participants were required to read the text message whilst completing the gait task and upon completion of the trial, relay the message back to the experimenter. Sentences ranged in length from 23 to 38 (31±6 mean ± SD) characters, including spaces (e.g. ‘Shall we go for pizza?’ or ‘Are you watching the football tonight?’). At the end of the trial, participants were required to relay the message back to the experimenter to ensure they had correctly engaged with the phone task.
4. Write and send a text message–Prior to the start of the trial, the experimenter told the participant what message to write on their phone. The participant verbally repeated what they were required to write, confirming they had heard the message correctly. Participants were required to write the text message whilst completing the gait task and send the text (to the experimenter’s phone) prior to completing the trial. At the end of the trial, the experimenter read their phone to confirm that the participant has sent the message correctly. The texting keyboard was the typical QWERTY keyboard. Predictive text was permitted. Sentences ranged in length from 15 to 36 (27±8 mean ± SD) characters, including spaces (e.g. ‘Shall we meet at the train station?’ or ‘What shall we do today?’).

The content for the *talk*, *read* and *write* phone conditions was similar in length and complexity to previous research e.g. [13,14]. Within the *talk*, *read* and *write* condition, participants were instructed to only start using their phone after they had turned around and started walking.

Of note, trials were the participant had completed the task (*talk*, *read or write*) on their phone prior to negotiating the surface height change were disregarded and repeated using a longer sentence. This only occurred twice for one participant.

### Equipment

Visual search behaviour was recorded using an SMI iViewETG head mounted mobile eye tracker (SensoMotoric Instruments Inc, Warthestr; Germany, Ver. 1.0) and was sampled at 30 Hz. The eye tracker contains 3 cameras built into the glasses, an infrared camera to record movements of each eye and a high definition camera (24 Hz) to record the visual scene. The eye tracker also contains an integrated microphone to record audio. Data from the eye tracker were recorded on a mini laptop (Lenovo X220, ThinkPad, USA) with iView ETG (Ver. 2.0) recording software installed. A three point eye calibration was performed to verify point-of-gaze and the calibration was checked following every third trial. The spatial resolution of the system was 0.1°, with gaze position accuracy of ±0.5°. The laptop was placed in a backpack which was worn by the participant during testing. None of the participants reported that wearing the backpack affected their balance whilst walking.

Three-dimensional kinematic data were sampled at 100 Hz using a motion capture system (Codamotion movement analysis system; Charnwood Dynamics Ltd, UK). Three coda units were positioned around the laboratory to create a 360° capture volume as the participant negotiated the surface height change. Active markers were attached bilaterally, to the superior aspects of the second metatarsal head, the most distal, superior aspect of the second toe, the lateral malleoli, the posterior aspect of the calcanei, the sternum and antero-lateral and postero-lateral aspects of the head.

Electronic timing gates (Smart-Speed, Fusion Sport, Australia) were positioned at the start and end of the 5.6m walkway. As the participant walked past the timing gates, a single ‘beep’ was emitted. The auditory tone recorded by the eye tracker provided the trial length and start and end points to begin tracking the visual search data.

### Data analysis

#### Visual search

Point of gaze data from the eye tracker was analysed offline using BeGaze software (Ver.3.4) and was subject to frame by frame analysis. Each trial was tracked from the first frame the auditory noise from the light gates (denoting the start of the trial) was registered on the eye tracker’s microphone up until the second auditory noise from the light gates was registered (denoting the trial being complete).

Areas of interest (AOI) were used to define key locations within the visual scene and comprised of; Phone, Intended travel path, Surface height change and ‘‘Other” (denoting fixations to task-irrelevant locations within the display). Intended travel path was defined as the area down in front of the participant on the walkway and when looking straight ahead to provide information regarding direction of travel. Each point of gaze in the real-time dynamic visual scene was mapped manually (frame by frame) to the AOIs. Fixations were determined as four or more consecutive frames (120 ms) to an area of interest; a threshold consistent with previous research used to define a fixation (e.g. [31]).

The following variables were used to analyse eye tracking data;

1. Trial length–see description above.
2. Relative number of fixations on each AOI–Higher number of fixations to a particular AOI provides an indication of the AOI’s relative relevance to information processing and subsequent task execution [32]. Calculated as a percentage overall trial length to account for any differences in actual trial length between conditions.
3. Relative fixation time on each AOI–Longer time spent fixating at a particular AOI allows more information to be obtained, indicating greater relevance to information processing and subsequent task execution [32]. Calculated as a percentage overall trial length to account for any differences in actual trial length between conditions.

#### Gait

**Fig 2:**
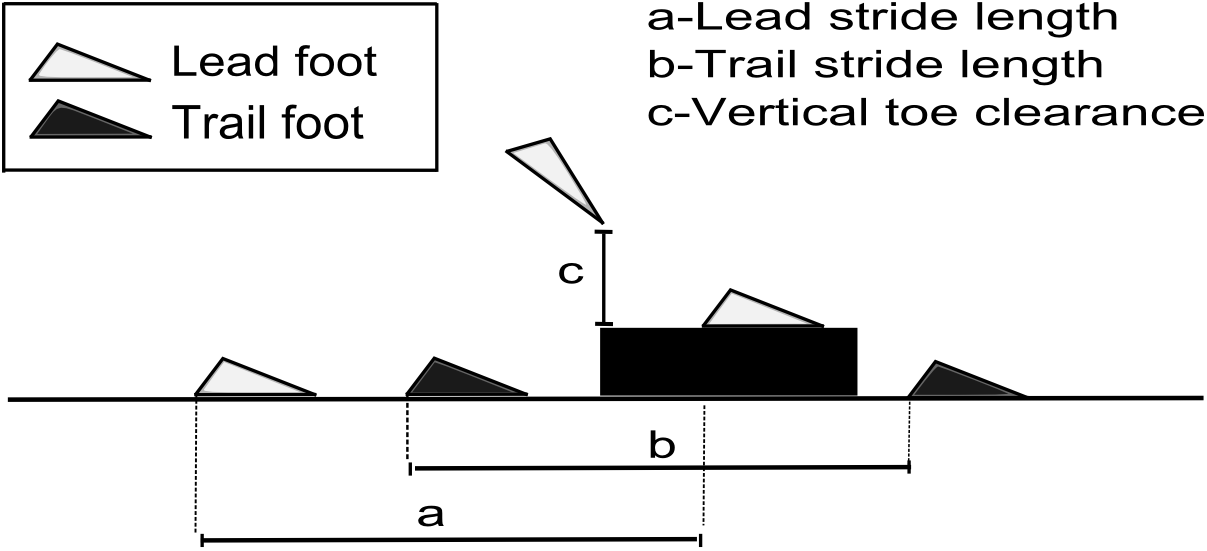
Representation of foot placement and clearance parameters for the lead and trail foot during negotiation of the surface height change.

Negotiation of the initial obstacle was not analysed. The obstacle was placed in the walkway in an attempt to make the task increasingly realistic since in everyday life we frequently negotiate multiple obstacles in our travel path. Analysis focussed on how both left and right limbs were positioned on the floor in the final step immediately prior to negotiating the surface height change, negotiation of the surface height change and the placement of each foot after crossing the surface height change (Fig 2). Due to the foot (left or right) participants initiated gait with at the start of the trial altering throughout the study, the lead foot which stepped up onto the surface height change was not consistent. Therefore, the following variables were not assigned to each (left or right) limb, rather ‘lead’ and ‘trail’ limb to reflect the first and second foot stepping up onto the surface height change. The following variables were analysed as they are considered important to assess the kinematics of gait and changes in such variables have been shown to increase the risk of tripping / falling [33];

1. Lead and trail limb foot position prior to crossing the rising edge of the surface height change. Calculated as the anterior-posterior distance between the foot and the front upper edge of the surface height change.
2. Lead and trail foot stride length–anterior-posterior distance between the lead foot when planted on the floor (final step prior to negotiating surface height change) and when planted on the surface height change (lead foot stride length). Anterior-posterior distance between the trail foot when planted on the floor (final step prior to negotiating surface height change) and when planted on the floor after negotiating the surface height change (trail foot stride length, Fig 2).
3. Lead and trail limb vertical toe clearance–Vertical distance between the upper edge of the surface height change and toe as it crosses the rising edge of the surface height change (Fig 2).
4. Lead and trail limb horizontal toe velocity—Calculated at the point of vertical toe clearance (lead and trail limb respectively).
5. Lead and trail limb foot position after crossing the rising edge of the surface height change. Calculated as the anterior-posterior distance between the foot and the front upper edge of the surface height change.
6. Head Flexion. Head flexion was calculated at penultimate foot contact with the floor, final foot contact with the floor (both prior to negotiating the surface height change), instance of lead vertical toe clearance, and at lead and trail foot contact with the floor after crossing the surface height change. Head flexion was normalized such that 0° was looking straight ahead. Positive head flexion indicated looking down towards the ground, and negative head flexion indicated looking up towards the ceiling.
7. Medial-lateral bivariate variable error (BVE). Medial-lateral bivariate variable error (BVE) was calculated to determine how much each participant deviated from a straight travel path during each condition. For each trial, BVE was calculated as the difference between the absolute medial-lateral distance of the sternum marker for each frame (xi) to the mean position during the trial (xm);
  $$Medial-lateral\;bivariate\;error=\sqrt{(\frac{1}{n})\sum_{i=1}^{n}{\left(x_{i}-x_{m}\right)}^{2}}$$

### Statistical design

All participants completed each phone condition (*no phone*, *talk*, *read*, and *write*), three times, in a fully randomised order. For the analysis of visual search data, from the 21 participants collected, 4 participants were excluded from the analysis due to a tracking ratio below 90% [34]. The tracking ratio considers the number of video frames whereby no data was recorded by the eye tracker (i.e. ability to track eye movement was lost). A higher tracking ratio means that more frames were recorded (conversely, fewer frames relating to eye movements were lost). An acceptable tracking ratio of ≥90% was used to ensure that only reliable eye-tracking data were included. All 21 participants were included in the gait analysis.

For the analysis of visual search data, only the first trial among the three at a given phone condition was used whereby a participant recorded a tracking ratio above 90% and good pre-post calibration [34]. This resulted in a total of 68 observations (17 participants x 4 phone conditions x 1 occurrence) being included for statistical analysis.

A random selection of trials from five participants (29%) was coded by two researchers (KvP and HB) to assess gaze behaviour inter-rater reliability. An acceptable average intraclass correlation coefficient of *r* = 0.89 was reached for the frame by frame mapping of time looking at the phone (*r* = 0.99), surface height change (*r* = 0.86) and intended travel path (*r* = 0.93).

A total of 252 observations were recorded from the analysis of gait data (21 participants x 4 phone conditions x 3 occurrences), however, because the analysis of gait trials was an average across the three occurrences (trial repeats), 84 data values were utilised for statistical analysis. To ascertain whether the consistency with which the surface height change was positioned from the start position affected results, analysis was conducted on the repetition effect and between the time taken to complete the first, twelfth (mid) and final (last) trial.

Levene’s test for equal variance and the Kolmogorov-Smirnov test confirmed equal variance and normality of the data (p > 0.05). Data were analysed using a one-way repeated measures ANOVA with phone (no phone, write, read, and talk) as the independent variable. Level of significance was accepted at p < 0.05. Post-hoc analysis where appropriate was completed using pairwise comparisons (Bonferroni correction). Effect sizes were calculated using Partial Eta squared ƞ_p_^2^.

## Results

### Visual search

There was a significant effect of phone condition on trial time (p < .001). Trial time was significantly longer in the *write* compared to all other conditions (Table 1). The increase in trial time in *write* compared to *read*, *talk* and *no phone* conditions represented a 67%, 83% and 118% change respectively. Trial time was also significantly longer in *read* and *talk* compared to *no phone* (31% change and 19% change for *read* and *talk* respectively) and *read* was significantly longer than *talk* (10% change).

**Table 1:** Visual search parameters as a function of phone task. Data presented are the group mean (standard deviation).

	No phone	Talk	Read	Write	P value	n_p_^2^
**Trial Time (s)**	4.79	5.71	6.27	10.46	p<0.001	.714
	(.64)	(.57)	(1.37)	(3.23)		
**No. fixations (%)**						
**Phone**	-	-	37.83	55.34	p < .05	.730*
			(23.27)	(24.75)		
**Surface Height Change**	17.07	10.24	6.73	6.77	p < .05	.189
	(14.52)	(7.42)	(6.58)	(11.06)		
**Intended Travel Path**	51.16	37.35	28.37	25	p<0.001	.375
	(19.20)	(13.86)	(17.33)	(18.60)		
**Other**	29.74	49.22	23.05	9.62	p<0.001	.475
	(25.50)	(21.56)	(22.20)	(13.81)		
**Fixation time (%)**						
**Phone**	-	-	60.97	88.18	p<0.001	1.51*
			(21.57)	(13.47)		
**Surface Height Change**	16.95	9.74	3.55	1.51	p<0.001	.436
	(13.71)	(9.46)	(5.36)	(2.22)		
**Intended Travel Path**	57.02	46.49	18.02	6.3	p<0.001	.745
	(20.29)	(15.24)	(14.94)	(9.52)		
**Other**	16.25	28.38	8.14	0.01	p<0.001	.467
	(20.86)	(18.94)	(11.02)	(.01)		

NB. ‘-‘ indicates no fixation.

* denotes effect size calculated using Cohen’s *d*.

#### Relative number of fixations

Statistical analysis of the relative number of fixations on the phone in *no phone* and *talk* conditions are not reported due to this area of interest not being present during the trial, subsequently resulting in comparison between these conditions being redundant. There were significantly more fixations (46% increase) on the phone in the *write* compared to *read* condition (p < .05, Table 1).

**Fig 3:**
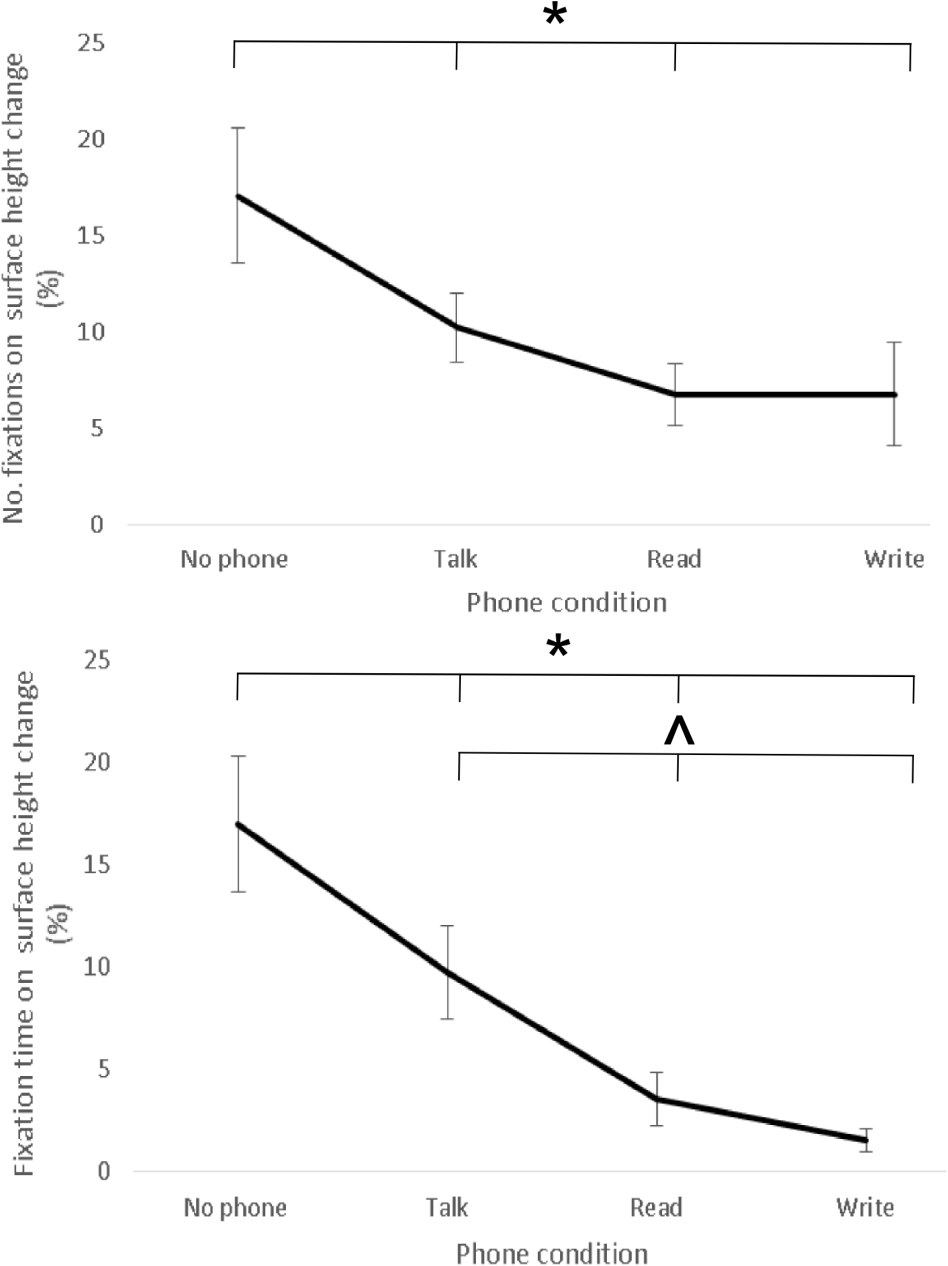
Relative number (a, top figure) and length (b, bottom figure) of fixations at the surface height change in each phone condition (group mean ± SE). Fig 3 top and bottom—No phone is significantly different to talk, read and write conditions. Fig 3 bottom—Talk is also significantly different to read and write conditions.

There was a significant effect of phone condition on the relative number of fixations on the surface height change (p *<* .05) and intended travel path, (p < .001). Post hoc analysis showed that significantly more fixations were made to the surface height change (Fig 3A) and intended travel path in the *no phone* compared to all other conditions (Table 1). The increase in number of fixations at the surface height change in *no phone* compared to *talk*, *read* and *write* conditions represented a 40%, 61% and 60% change respectively and at the intended travel path in *no phone* compared to *talk*, *read* and *write* conditions represented a 27%, 45% and 51% change respectively. There were also significantly more fixations (33% increase) on the intended travel path in the *talk* compared to the *write* condition.

There was a significant effect of phone condition on the relative number of fixations on Other locations (p *<* .001). There were significantly more fixations on Other locations in *talk* compared to *no phone*, *read* and *write* conditions (Table 1). The increase in number of fixations at other task-irrelevant locations in *talk* compared to *no phone*, *read* and *write* conditions represented a 40%, 53% and 80% change respectively. There were significantly more fixations on Other locations in the *no phone* (68% increase) and *read* (58% increase) compared to *write* condition.

#### Relative fixation time

Statistical analysis of the relative fixation time on the phone in *no phone* and *talk* conditions are not reported due to this area of interest not being present during the trial, subsequently resulting in comparison between these conditions being redundant. There was significantly longer relative fixation time (45% increase) on the phone in the *write* compared to *read* condition (p < .001, Table 1).

There was a significant effect of phone condition on the relative fixation time on the surface height change (p < .001). There was significantly longer relative fixation time on the surface height change in the *no phone* compared to *talk*, *read* and *write* conditions (Fig 3B). The increase in fixation time at the surface height change in *no phone* compared to *talk*, *read* and *write* conditions represented a 43%, 79% and 91% change respectively. There was significantly longer relative fixation time on the surface height change in the *talk* compared to *read* and *write* conditions, representing a 64% and 84% increase in *talk* compared to *read* and *write* respectively.

A significant effect of phone condition on the relative fixation time on the intended travel path was found (p < .001). There was significantly longer fixation time on the travel path in the *no phone* and *talk* compared to both *read* and *write* conditions. The increase in fixation time on the intended travel path in the *no phone* was 68% and 89% higher compared to *read* and *write* respectively. The increase in fixation time in the *talk* condition was 61% and 86% higher compared to *read* and *write* respectively. There was significantly longer fixation time (65% increase) on the intended travel path in the *read* compared to *write* condition.

There was a significant effect of phone condition on the relative fixation time on Other task-irrelevant locations (p *<* .001). Post hoc analysis showed that there was significantly longer fixation time on Other in the *talk* compared to *no phone*, *read* and *write* conditions (Table 1). The increase in fixation time in *talk* compared to *no phone*, *read* and *write* conditions represents a 43%, 71% and 100% change respectively. There was significantly longer fixation time on Other in the *no phone* and *read* compared to *write* condition. The increase in fixation time in the *no phone* and *read* condition was 100% higher (both conditions) compared to *write*.

### Gait

There were no trips or contacts made with the surface height change by any participant throughout the study. There was no significant effect of repetition on any of the gait variables (p > .05). Furthermore, there was no significant effect of trial number on time to complete the trial (p = .418). Average trial time for the first, mid and final trials were 7.51 ± 3.65s, 6.53 ± 2.33s and 7.13 ± 3.01s respectively.

Prior to crossing, there was a significant effect of phone condition on lead limb foot position in relation to the front edge of the surface height change (p < .001). The foot was positioned significantly closer to the front edge of the surface height change in write compared to no phone, talk and read conditions and represented a 37%, 24% and 21% change respectively. Prior to crossing, lead limb foot position was significantly closer to the front edge of the surface height change in the read compared to no phone condition, representing an 18% change.

Prior to crossing, there was a significant effect of phone condition on trail limb foot position in relation to the front edge of the surface height change (p < .001). The foot was positioned significantly closer to the front edge of the surface height change in write compared to no phone, talk and read conditions and represented an 11%, 11% and 3% change respectively. Prior to crossing, trail limb foot position was significantly closer to the front edge of the surface height change in the read compared to no phone and talk condition, representing an 8% change for both conditions.

There was a significant effect of phone condition on lead stride length (p < .001) and trail stride length (p < .001). Both lead and trail stride length was significantly shorter in *write* compared to all other conditions (Table 2). The reduction in lead stride length in *write* compared to *no phone*, *talk* and *read* conditions represented a 38%, 28% and 26% change respectively. In trail stride length, *write* compared to *no phone*, *talk* and *read* conditions represented a 44%, 35% and 36% change respectively. Lead stride length was also significantly shorter in *talk* and *read* compared to *no phone*, which represented a 14% and 16% decrease in *talk* and *read* (respectively) when compared to *no phone* condition. Trail stride length was significantly shorter in *talk* (15% reduction) and *read* (13% reduction) compared to *no phone* condition.

**Table 2:** Gait parameters as a function of phone task condition. Data presented are the group mean (standard deviation).

	No Phone	Talk	Read	Write	P value	n_p_^2^
**Lead limb vertical toe clearance (mm)**	120	123	127	142	p<0.001	.295
	(22)	(23)	(30)	(28)		
**Lead limb horizontal toe velocity (m.s**^**-1**^**)**	3.84	3.15	3.4	2.32	p<0.001	.731
	(.51)	(.53)	(.46)	(.59)		
**Trail limb vertical toe clearance (mm)**	84	87	97	90	p = .025	.150
	(21)	(23)	(28)	(28)		
**Trail limb horizontal toe velocity (m.s**^**-1**^**)**	3.09	2.61	2.61	1.91	p<0.001	.638
	(.67)	(.44)	(.39)	(.41)		
**M/L BVE (m)**	0.57	0.72	0.61	1.35	p<0.001	.361
	(.50)	(.73)	(.44)	(.73)		
**Lead step length (m)**	1.29	1.11	1.08	0.8	p<0.001	.690
	(.14)	(.13)	(.17)	(.18)		
**Trail step length (m)**	1.26	1.07	1.09	0.7	p<0.001	.751
	(.15)	(.17)	(.17)	(.25)		
**Lead limb foot position (m)**	0.22	0.14	0.21	0.13	p<0.001	.399
	(.07)	(.08)	(.07)	(.08)		
**Trail limb foot position (m)**	0.94	0.75	0.84	0.49	p<0.001	.694
	(.11)	(.17)	(.16)	(.23)		
**Head angle (°)**						
**Penultimate foot contact**	1	2	22	32	p<0.001	.725
	(9)	(12)	(15)	(11)		
**Final foot contact**	-1	0	17	34	p<0.001	.670
	(11)	(15)	(19)	(13)		
**Instance of lead toe clearance**	2	1	20	32	p<0.001	.680
	(11)	(14)	(15)	(11)		
**Lead foot contact after crossing**	0	1	18	32	p<0.001	.188
	(11)	(14)	(17)	(11)		
**Trail foot contact after crossing**	-10	-7	7	31	p<0.001	.764
	-9	-12	-16	-12		

NB. Head angle was normalised such that 0° indicates looking straight ahead. Negative head angle values indicate looking up and positive values looking down. Negative foot placement values indicate placement prior to the front rising edge; a larger negative value indicates further away from the front rising edge

**Fig 4:**
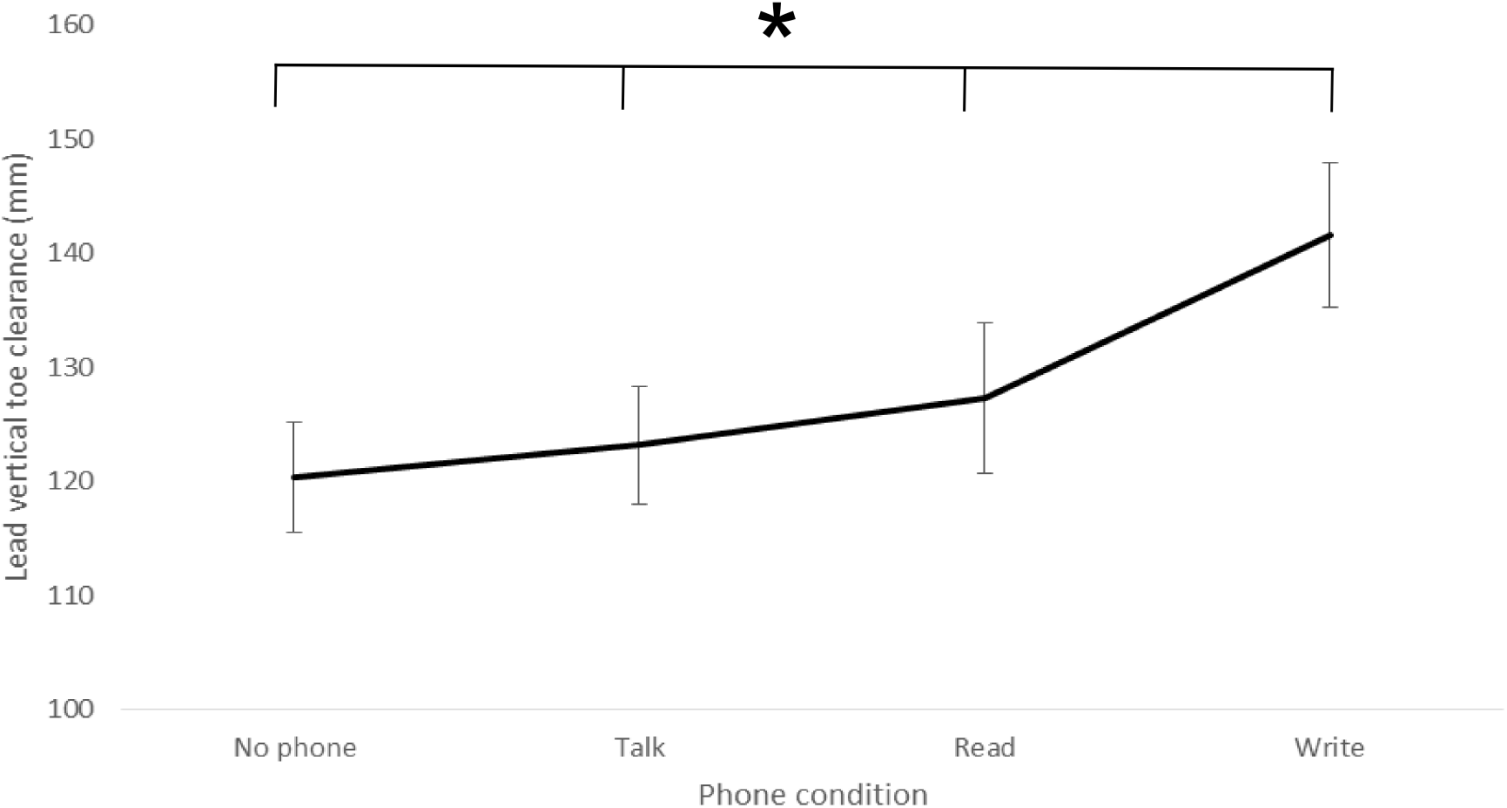
Lead vertical toe clearance when negotiating the surface height change under different phone conditions (group mean ± SE). Write condition is significantly different to *no phone*, *talk* and *read* conditions.

There was a significant effect of phone condition on lead toe clearance (p < .001), lead toe crossing velocity (p < .001) and trail toe crossing velocity (p < .001, Table 2). Post hoc analysis indicated that the lead foot was lifted significantly higher and both lead and trail foot significantly slower over the edge of the surface height change in *write* compared to all other conditions (Table 2, Fig 4). The increase in lead toe clearance in *write* compared to *no phone*, *talk* and *read* conditions represented an 18%, 15% and 12% change respectively. The reduction in lead toe crossing velocity in *write* compared to *no phone*, *talk* and *read* conditions represents a 40%, 26% and 32% change respectively. The reduction in trail toe crossing velocity in *write* compared to *no phone*, *talk* and *read* conditions represented a 38%, 27% and 27% change respectively. Lead toe crossing velocity was also significantly slower in *talk* compared to *no phone* and *read* condition, representing a 22% and 8% reduction in *talk* when compared to *no phone* and *read* conditions respectively. Trail toe crossing velocity was also significantly slower in the *read* (16% reduction) and *talk* (16% reduction) compared to *no phone* condition (Table 2).

There was a significant effect of phone condition on trail toe clearance (p = .025). The trail foot was lifted significantly higher in *read* compared to *no phone* and *talk* conditions (Table 2). This represented a 15% and 11% increase in *read* when compared to *no phone* and *talk* conditions respectively.

After crossing, there was a significant effect of phone condition on lead limb foot position in relation to the front edge of the surface height change (p < .001). The foot was positioned significantly closer to the front edge of the surface height change in *write* and *talk* compared to *no phone* and *read* conditions (write compared to *no phone* and *read*, 41% change and 38% change respectively; *talk* compared to *no phone* and *read*, 36% change and 50% change respectively).

After crossing, there was a significant effect of phone condition on trail limb foot position in relation to the front edge of the surface height change (p < .001). The foot was positioned significantly closer to the front edge of the surface height change in *write*, *talk* and *read* compared to *no phone* condition (Table 2). The reduction in foot position in *write*, *talk* and *read* compared to *no phone* condition represented a 48%, 20% and 11% change respectively. Trail limb foot position was also significantly closer to the front step edge in *write* (42% change) and *talk* (11% change) compared to *read*. The foot was also significantly closer in *write* compared to *talk* condition (35% change).

There was a significant effect of phone condition on head angle (p < .01). At each instance in the movement, the head was significantly more flexed in *write* compared to all other conditions (Table 2). The head was also significantly more flexed in the *read* compared to *no phone* and *talk* conditions at penultimate foot contact with the floor, final foot contact with the floor and the instance of lead vertical toe clearance (Table 2).

There was a significant effect of phone condition on medial-lateral BVE (p < .001). There was significantly greater medial-lateral variability in the *write* compared to all other conditions (Table 2). The increase in medial-lateral BVE in *write* compared to *no phone*, *talk* and *read* conditions represented a 137%, 88% and 121% change respectively.

## Discussion

Pedestrians regularly engage with their mobile phone in a variety of ways whilst walking. Previous research has considered (separately) the impact of mobile phone use on visual search behaviour and walking gait. However, to date, there is no published research collectively investigating how mobile phone use impacts visual search behaviour and adaptive gait, when required to walk up to and safely negotiate a floor based obstacle. Findings from the current study illustrate clear differences visual search behaviour and gait when using a mobile phone compared to when not using a phone. Using a phone, compared to not using a phone, resulted in looking less frequently and for less time at the surface height change, and negotiating the surface height change in a manner consistent with adopting an increasingly cautious stepping strategy. Key differences in visual search and gait were also observed dependent upon the manner in which the phone was being used.

### Phone vs. no phone comparison

Results from the current study support previous findings highlighting that whilst walking, compared to no phone being present, using a phone (either reading a text, typing a text or talking) results in increased trial time / reduced walking speed [3,12–14,16]. However, we extend previous research to demonstrate that this is also evident when negotiating a surface height change. The current research also shows that when using a phone, increased trial time (and by implication reduced walking speed) was attributed to participants reducing both lead and trail foot stride length (due to altered foot placement pre / post surface height crossing) and has previously been shown to decrease as walking speed reduces e.g. [35] and lifting the feet higher and slower when negotiating the edge of the surface height change (Table 2, Fig 4).

Previous research has attributed reductions in walking speed to a combination of dual-task interference within the working memory [13], information processing ability [36], an attempt to lower the cognitive demands of the primary (walking) task [37,38] or an effect of reduced attention to walking [37]. Walking slower facilitates additional time to identify potential hazards and plan/initiate a suitable response (i.e. walk round the object or step over the obstacle) to minimise the risk of injury. However, it is important to recognise that walking slower when engaged with the mobile phone may not necessarily reduce the risk of pedestrian injury if they fail to ‘see’ the hazard in the first place, especially if the hazard suddenly appears in the environment (i.e. pedestrian walks across the travel path). Indeed, in the *write*, *read* and *talk* conditions, significantly fewer fixations were made to the environment (surface height change and intended travel path, Fig 3A, Table 1) compared to *no phone* condition. If the pedestrian does not re-orientate fixation towards the environment frequently enough, this may increase the risk of accident as potential hazards will not be seen early enough to allow time to plan/initiate a suitable avoidance response [17–19].

Interestingly though, the reduction in the relative number of fixations at the surface height change ranged from 40–61% and the intended travel path ranged from 27–51% when using the phone compared to no phone condition. Furthermore, in some instances in the current study, in *write*, *read*, and *talk* conditions, throughout the entire trial participants did not look directly at the surface height change. Despite this significant reduction in frequency (and length) of fixation to the environment, participants were able to safely negotiate the environment/hazards placed in the travel path. Previous research has shown that effective and safe travel is possible when a large proportion of time (~40%) is spent fixating at task irrelevant objects [20] and without having to reorientate fixation to directly look at key areas/object of interest. For example, the use of peripheral vision can be effective in safely stepping over an obstacle [21] or up onto a step [22] and when using a mobile phone, navigate whilst cycling [23] driving [24,25] and walking [14,16]. Indeed, Ahlstrom et al. [23] highlighted that whilst cycling outdoor and engaging with a phone, individuals had fixations to the phone frequently exceeding 5 seconds and in some instances reaching ~20 seconds. Despite these long periods whereby no visual information was acquired from the road using the fovea (central vision), cyclists did not crash. This led Ahlstrom et al. [23] to suggest that cyclists were able to effectively rely on peripheral vision for guidance while their fovea was directed at the phone. In the current study, no contacts with the surface height change were observed, indicating that concurrent visual information from the fovea may not be necessary to safely negotiate complex terrain whilst engaging with a mobile phone. It is likely that participants use a combination of central and peripheral vision to navigate their travel path when interacting with a mobile phone.

A possible mechanism to adapt to the task when stepping up onto the surface height change, compared to the *no phone* condition, were that participants lifted the lead and / or trail foot significantly higher over the edge of the surface height change and / or crossed the edge with significantly slower horizontal foot velocity (Table 2, Fig 4). Increasing foot clearance over the obstacle provides a greater margin for error, subsequently reducing the risk of contacting the surface height change. Reducing crossing velocity minimises the risk of tripping if contact is made with the surface height change edge, since any contact will have less effect on perturbing balance [18]. These adaptations in the movement indicate a cautious stepping strategy, which may be the result of two factors. The significant reduction in time spent looking at the surface height change when using the mobile phone subsequently meant that participants were unable to acquire a precise visual representation or accurately perceive the obstacle’s characteristics prior to stepping up. Alternatively, participants were required to rely on information from the peripheral visual field, which has previously been shown to provide poorer resolution of fine detail (visual acuity, [39]) and spatial modulation sensitivity [40] compared to central vision.

The increase in medial-lateral deviation when using the phone may indicate an increased risk of injury compared to not using a phone. When walking up to and negotiating the surface height change, participants demonstrated greater medial-lateral deviation in *write* compared to the *no phone* condition (*write* was also significantly different to *read* and *talk* conditions, Table 2). Increased medial-lateral deviation when walking poses implications for pedestrian safety since deviating from the intended travel path increases the risk of colliding into oncoming pedestrians and objects [3], which is a leading cause of mobile phone related pedestrian injury [41].

Increased medial-lateral deviation in the *write* condition compared to the *no phone* (and indeed *read* and *talk* conditions) is likely attributed to the associated changes in head flexion and visual search behaviour. In the *write* condition, participants significantly increased head flexion and looked down at the phone for longer and more frequently compared to all other conditions (Tables 1 and 2). The increased visual attention to the mobile phone resulted in reduced fixation time at the intended travel path in the *write* condition compared to the *no phone* (and *read* and *talk*) conditions. Because of this, participants may not have been able to acquire sufficient visual information using the central visual field to plan direction of travel. Alternatively, due to the position of the head (increased flexion) it is possible that the peripheral visual field was unable to acquire adequate visual information regarding path planning.

When using the mobile phone, compared to the no phone condition, the lead foot was positioned significantly closer to the front edge of the surface height change after stepping up onto the upper level (ranging from 36–41% change, Table 2). Inappropriate foot placement when stepping up to a new level will increase the risk of falling since the position of the foot determines the quality of the base of support for the weight-bearing phase whereby only that foot is in contact with the ground [29]. However, in the current study, despite the foot being positioned closer to the front surface height change, the entire foot was always safely positioned on the step (i.e. the heel was never over hanging the front edge of the step). This findings does not suggest reduced stability / increased risk of falling, rather likely reflects the reduced stride length observed (Table 2) with walking slower.

### Within phone comparison

When engaging with the mobile phone, the greatest adaption in gait strategy was observed in the *write* compared to the *read* and *talk* conditions. In the *write* condition, when stepping up onto the surface height change, participants lifted their lead foot significantly higher over the edge of the surface height change and crossed with a slower lead and trail foot velocity compared to the *read* and *talk* conditions (Table 2, Fig 4). Participants also reduced their stride length, walked slower (increased trial length) and demonstrated greater medial-lateral deviation. Adaptations in gait strategy was also observed in *read* compared to *talk* condition (Table 2). Some of these adaptations have been discussed in the previous sub-section.

The differences observed in walking gait in the *write* and *read* compared to *talk* condition may be attributed to the type of information relied on in the secondary task. Completing the walking task would have predominantly relied on the visual-spatial resources of the working memory. The *read* and *write* conditions would have also relied on the same visual-spatial resources of the working memory, whereas the *talk* condition would have likely drawn on the more distinct resource from the phonological loop. The greater changes in gait in the *write* and *read* compared to *talk* condition may be attributed to the utilisation of the same working memory resources as opposed to the sharing of working memory demands in the *talk* condition [42–45]. In the *write* condition, the added motor demands required for reading and writing the text message, in addition to the cognitive processes required for the communication interchanges [26] may explain why *write* had the greatest effect on walking gait when compared to *read* condition. Furthermore, writing a text may increase the visual attention demands required to locate the keypad to type the text in addition to confirming (reading) what has been written on the screen is correct [42]: which may explain why the number and duration of fixations at the phone was greatest in this condition (Table 1).

In the *write* and *read* condition, throughout the trial a large proportion of visual attention was directed towards the phone (~88±13% and ~61±22% in *write* and *read* conditions respectively, Table 1). Whilst the proportion of visual attention being directed towards the phone was greater in the *read* compared to *talk* and *no phone* conditions (where the phone was not present in the visual field), this was significantly less than the *write* condition. The reduction in time spent fixating at the phone in the *read* compared to *write* condition facilitated additional time throughout the trial to fixate at the intended travel path direction (~6±10% and ~18±15% in *write* and *read* conditions respectively, Table 1). The additional visual information acquired from the intended travel path may explain why in the *read* condition, medial-lateral deviation was significantly less than the *write* condition and similar compared to the *no phone* and *talk* condition. Furthermore, with the head being less flexed (i.e. looking up more within the environment) in the *read* compared to the *write* condition (Table 2), this facilitated the use of the superior peripheral visual field to provide directional guidance via optic flow [46]; the complex flow pattern of visual motion at the retina.

It is important to emphasise that the results from the current study do not provide a direct indication of why mobile phone use has been reported to increase the risk of pedestrian related accidents (e.g. [9]). Generally, the present findings actually indicate a more cautious strategy when negotiating the surface height change located within the environment. It is therefore likely that pedestrian related accidents when engaged with a mobile phone are attributed to tasks that require greater attentional demands and thus provide a greater demand on working memory; such as walking down the street where you are required to attend to numerous potential hazards, crossing the road when you are required to attend to oncoming hazards (fellow pedestrians or cars) travelling at different velocities e.g. [27] or when the phone interaction is increasingly demanding i.e. a particularly engaging/important conversation.

## Limitations

The limitation with using the eye tracker is the assumption that people are attending to where they are looking. Research has shown that it is possible to ‘shift’ our attention without moving our eyes [47]; pedestrians may not be perceiving information from the precise location where they are looking. However, with complex stimuli such as walking in the environment, research has shown that it is more efficient to move our eyes than move attention [48,49]. Conversely, it is possible that participants were looking at key areas within the environment (e.g. surface height change) but failing to perceive or process the obstacle’s characteristics. Indeed, Stavrinos et al., [27] identified that in a virtual street crossing task, when *talk*ing on the phone, pedestrians appeared to be engaging in appropriate precautionary measures prior to crossing the road (turning their head from side-to-side to inspect oncoming traffic), but were still involved in significantly more virtual accidents (i.e. hit by a vehicle) compared to crossing without a phone, which was hypothesised as a result of failure to detect or process critical information pertaining to oncoming vehicles.

In the current study, participants completed the task in a stable environment. The results from the current study suggest that pedestrian related accidents when using a mobile phone are likely attributed to unexpected changes in the environment not being seen or reacted to by the pedestrian. Indeed, an unexpected change in the environment should produce a reorientation in attention [50,51], however, if attention is currently focussed on the mobile phone or distracted somewhere else [52], this reorientation in attention may either not occur, or occur too late to avoid accident. In real world situations (i.e. outside of a laboratory testing environment) pedestrians face obstacles which they will have seen several steps in advance (e.g. a kerb or step), to something they may not have seen or has suddenly appeared during the concurrent step (e.g. another pedestrian changing direction and walking across their path). Our study adopted a stable scenario whereby the obstacle and surface height change were presented several steps in advance of the participant. This is similar to the approach used in other walking based studies e.g. [13,14,37]. Future research should consider investigating the impact of mobile phone use during a more dynamic environment as this would probably elicit a different response in gait and visual search to that observed in the current study. However, this future research direction should be considered separately to the findings reported in the present study due to the extent this occurs in real world situations (i.e. obstacles rarely appear in the travel path).

In the current study, participants negotiated the surface height change leading with either the left or right limb. Previous research has demonstrated that gait asymmetry can influence the kinematics of level walking and obstacle crossing e.g. [53,54]. In the present study, limb dominance was not recorded from participants, resulting in being unable to aggregate the data accounting for foot dominance when negotiating the surface height change. Whilst the impact of gait asymmetry may act as a confounding influence within the present results, it is relevant to note that gait asymmetry has predominantly been reported among older adults (the present study used young healthy adults) due to muscle imbalance in the leg e.g. [55,56]. Furthermore, the strength of the significance and reported percentage differences between mobile phone conditions for lead and trail limb variables indicates that we can be confident of the findings.

## Conclusion

The current study investigated how mobile phone use affects pedestrian’s visual search behaviour and gait when negotiating a floor based hazard in the travel path. Findings support our initial hypotheses, highlighting that pedestrians altered their visual search behaviour and adaptive gait when using their phone compared to no phone being present. Furthermore, tasks which required the individual to fixate predominantly at the phone’s screen had a greater effect on pedestrians’ visual search behaviour and adaptive gait. Specifically, compared to the *no phone* condition, adaptations in visual search behaviour and gait were observed when writing, reading or talking on the mobile phone. The adaptations in gait when negotiating the surface height change were consistent with participants adopting an increasingly cautious stepping strategy which may serve to reduce the risk of tripping / falling. Writing a text message whilst walking resulted in the greatest adaptions in visual search and gait compared to reading a text and talking on a mobile phone. Collectively, these findings indicate that mobile phone users adapt their visual search behaviour and gait to incorporate mobile phone use in a safe manner when negotiating static floor based obstacles.

## Supporting information

S1 File Raw gait and visual search data from each participant group in each test condition. (ZIP)

## References

[1] CTIA-The Wireless Association. CTIA–Advocacy. 2011. Available from: http://www.ctia.org.

[2] Duggan, M., Rainie, L. Cell phone activities 2012. Washington, DC: Pew Research Center. 2012. Available from: http://pewinternet.org/Reports/2012/Cell-Activities.aspx.

[3] Schabrun SM, van den Hoorn W, Moorcroft A, Greenland C, Hodges PW. Texting and walking: strategies for postural control and implications for safety. PLoS One. 2014; 22:9(1):e84312. 10.1371/journal.pone.0084312 PMC3898913 24465402

[4] Smith, A. The best (and worst) of mobile connectivity. Washington, DC: Pew Research Center. 2012. Available from http://pewinternet.org/Reports/2012/Best-Worst-Mobile.aspx.

[5] Thornton B., Faires A., Robbins M., Rollins E. The mere presence of a cell phone may be distracting: Implications for attention and task performance. Social Psychology. 2014; 45(6): 479–488.

[6] The Radicati Group. Email Statistics Report, 2013–2017. 2013. Available from: http://www.radicati.com/?p=9659.

[7] Pew Research Center. John Horrigan. Pew Internet and American Life Project. Info on the go: Mobile access to data and information. 2008. Available from: http://pewresearch.org/pubs/753/mobile-access-datainformation.

[8] Smith D.C., Schreiber K.M., Saltos A., Lichenstein S.B. and Lichenstein R. Ambulatory cell phone injuries in the United States: An emerging national concern. Journal of safety research. 2013; 47:19–23. doi: 10.1016/j.jsr.2013.08.003 24237866 10.1016/j.jsr.2013.08.003

[9] Nasar J.L., Troyer D. Pedestrian injuries due to mobile phone use in public places. Accident Analysis and Prevention. 2013; 57(0): 91–95. 23644536 10.1016/j.aap.2013.03.021

[10] GHSA. Everyone Walks. Understanding and addressing pedestrian safety. Governors Highway Safety Associations. 2015. Available from: http://www.ghsa.org/html/files/pubs/sfped.pdf.

[11] Sky UK. 'Go Slow' Smartphone Walking Lane For Dawdlers. 2015. Available from: http://news.sky.com/story/1336075/go-slow-smartphone-walking-lane-for-dawdlers.

[12] Hyman I. Jr, Boss M., Wise B., McKenzie K., Caggiano J. Did you see the unicycling clown? Inattentional blindness while walking and *talk*ing on a cell phone. Applied Cognitive Psychology. 2010; 24: 597–607.

[13] Lamberg E.M., Muratori L.M. Cell phones change the way we walk. Gait and Posture. 2012; 35: 688–690. doi: 10.1016/j.gaitpost.2011.12.005 22226937 10.1016/j.gaitpost.2011.12.005

[14] Lopresti-Goodman S. M., Rivera A., Dressel C. Practicing safe text: The impact of texting on walking behavior. Applied Cognitive Psychology. 2012; 26(4): 644–648.

[15] Nasar J., Hecht P., Wener R. Mobile telephones, distracted attention, and pedestrian safety. Accident Analysis and Prevention. 2008; 40: 69–75. doi: 10.1016/j.aap.2007.04.005 18215534 10.1016/j.aap.2007.04.005

[16] Plummer P., Apple S., Dowd C., Keith E. Texting and walking. Effect of environmental setting and task prioritization on dual-task interference in healthy young adults. Gait and Posture. 2015; 41: 46–51. doi: 10.1016/j.gaitpost.2014.08.007 25193796 10.1016/j.gaitpost.2014.08.007

[17] Patla A.E., Greig M. Any way you look at it, successful obstacle negotiation needs visually guided on-line foot placement regulation during the approach phase, Neuroscience Letters. 2006; 397: 110–114. doi: 10.1016/j.neulet.2005.12.016 16413969 10.1016/j.neulet.2005.12.016

[18] Patla A.E., Rietdyk S. Visual control of limb trajectory over obstacles during locomotion: effect of obstacle height and width. Gait and Posture. 1993; 1: 45–60.

[19] Patla A.E. Understanding the roles of vision in the control of human locomotion. Gait & Posture. 1997; 5(1): 54–69.

[20] Foulsham T., Walker E., Kingstone A. The where, what and when of gaze allocation in the lab and the natural environment. Vision Research. 2011; 51(17): 1920–1931. doi: 10.1016/j.visres.2011.07.002 21784095 10.1016/j.visres.2011.07.002

[21] Marigold D. S., Weerdesteyn V., Patla A. E., Duysens J. Keep looking ahead? Re-direction of visual fixation does not always occur during an unpredictable obstacle avoidance task. Experimental Brain Research. 2007; 176: 32–42. doi: 10.1007/s00221-006-0598-0 16819646 10.1007/s00221-006-0598-0

[22] Timmis M.A., Scarfe A.C. and Pardhan S. How does the extent of central visual field loss affect adaptive gait?. Gait & posture. 2016; 44: 55–60. 27004633 10.1016/j.gaitpost.2015.11.008

[23] Ahlstrom C, Kircher K, Thorslund B, Adell E. Bicyclists’ visual strategies when conducting self-paced vs. system-paced smartphone tasks in traffic. Transportation research part F: traffic psychology and behaviour. 2016; 41: 204–16.

[24] Lamble D., Laakso M., Summala H. Detection thresholds in car following situations and peripheral vision: Implications for positioning of visually demanding in-car displays. Ergonomics. 1999; 42(6): 807–815.

[25] Summala H., Nieminen T., Punto M. Maintaining lane position with peripheral vision during in-vehicle tasks. Human Factors. 1996; 38(3): 442–451.

[26] Schwebel D., Stavrinos D., Byington K., Davis T., O’Neal E., de Jong D. Distraction and pedestrian safety: how *talk*ing on the phone, texting, and listening to music impact crossing the street. Accident Analysis and Prevention. 2012; 45: 266–71. doi: 10.1016/j.aap.2011.07.011 22269509 10.1016/j.aap.2011.07.011 PMC3266515

[27] Stavrinos D., Byington K.W., Schwebel D.C. Distracted walking: cell phones increase injury risk for college pedestrians. Journal of Safety Research. 2011; 42: 101–107. doi: 10.1016/j.jsr.2011.01.004 21569892 10.1016/j.jsr.2011.01.004

[28] Chou L. S., Draganich L. F. Placing the trailing foot closer to an obstacle reduces flexion of the hip, knee, and ankle to increase the risk of tripping. Journal of biomechanics. 1998; 31(8): 685–691. 9796668 10.1016/s0021-9290(98)00081-5

[29] Simoneau GG, Cavanagh PR, Ulbrecht JS, Leibowitz HW, Tyrrell RA. The influence of visual factors on fall-related kinematic variables during stair descent by older women. J Gerontol. 1991; 46: M188–M195. 1940077 10.1093/geronj/46.6.m188

[30] Oulasvirta A, Tamminen S, Roto V, Kuorelahti J. Interaction in 4-second bursts: the fragmented nature of attentional resources in mobile HCI. InProceedings of the SIGCHI conference on Human factors in computing systems 2005; 919–928. ACM.

[31] Williams A.M., Davids K., Burwitz L., Williams J.G. Visual search strategies in experienced and inexperienced soccer players. Research Quarterly for Exercise and Sport. 1994; 6(2): 127–135. 10.1080/02701367.1994.10607607 8047704

[32] Mann D.T.Y., Williams A.M., Ward P., Janelle C.M. Perceptual cognitive expertise in sport: a meta-analysis. Journal of Sport and Exercise Psychology. 2007; 29: 457–478. 17968048 10.1123/jsep.29.4.457

[33] Rhea C,K., Rietdyjk S. Influence of an unexpected perturbation on adaptive gait behaviour. Gait Posture. 2011; 34: 439–441. doi: 10.1016/j.gaitpost.2011.06.011 21764314 10.1016/j.gaitpost.2011.06.011

[34] Vansteenkiste P, Van Hamme D, Veelaert P, Philippaerts R, Cardon G, Lenoir M. Cycling around a curve: the effect of cycling speed on steering and gaze behavior. PloS one. 2014; 9(7): e102792 doi: 10.1371/journal.pone.0102792 25068380 10.1371/journal.pone.0102792 PMC4113223

[35] Murray M.P., Kory R.C., Clarkson B.H., Sepic S.B. Comparison of free and fast speed walking patterns of normal men. American Journal of Physical Medicine and Rehabilitation. 1966; 45: 8–24. 5903893

[36] Ophir E, Nass C, Wagner AD. Cognitive control in media multitaskers. Proceedings of the National Academy of Sciences. 2009; 106(37): 15583–7. 10.1073/pnas.0903620106 PMC2747164 19706386

[37] Kao P-C., Higginson C.L., Seymour K., Kamerdze M., Higginson J.S. Walking stability during cell phone use in healthy adults, Gait and Posture. 2015; 41: 947–953. 10.1016/j.gaitpost.2015.03.347 PMC4414910 25890490

[38] Törnros J.E.B., Bolling A.K. Mobile phone use—Effects of handheld and handsfree phones on driving performance. Accident Analysis & Prevention. 2005; 37: 902–909. 15946638 10.1016/j.aap.2005.04.007

[39] Mandelbaum J, Sloan LL. Peripheral Visual Acuity*: With Special Reference to Scotopic Illumination. American Journal of Ophthalmology. 1947; 30(5): 581–8. 20241713

[40] Hilz R., Cavonius C. R. Functional organization of the peripheral retina: sensitivity to periodic stimuli. Vision Research. 1974; 14(12): 1333–1337. 4446364 10.1016/0042-6989(74)90006-6

[41] Richtel M. Forget gum. Walking and using phone is risky. The New York Times. 2010; 17.

[42] Baddeley A. Working memory. Science. 1992; 255(5044): 556–9. 1736359 10.1126/science.1736359

[43] Beauchet O., Dubost V., Aminian K., Gonthier R., Kressig R.W. Dual-task-related gait changes in the elderly: does the type of cognitive task matter? Journal of Motor Behaviour. 2005; 37: 259–264. 15967751

[44] Woollacott M., Shumway-Cook A. Attention and the control of posture and gait: a review of an emerging area of research. Gait and Posture. 2002; 16: 1–14. 12127181 10.1016/s0966-6362(01)00156-4

[45] Wrightson JG, Ross EZ, Smeeton NJ. The effect of cognitive-task type and walking speed on dual-task gait in healthy adults. Motor control. 2016; 20(1): 109–21. doi: 10.1123/mc.2014-0060 25823560 10.1123/mc.2014-0060

[46] Bardy B.G., Warren W. H., Kay B. A. The role of central and peripheral vision in postural control during walking. Perception & Psychophysics. 1999; 61: 1356–1368. 10572464 10.3758/bf03206186

[47] Posner M.L. Orienting of attention. Quarterly Journal of Experimental Psychology. 1980; 32: 3–25. 7367577 10.1080/00335558008248231

[48] He E., Kowler E. The role of saccades in the perception of texture patterns. Vision Research. 1992; 32: 2151–2163. 1304092 10.1016/0042-6989(92)90076-u

[49] Sclingensiepen K. H., Campbell E.W., Legge G.E., Walker T.D. The importance of eye movements in the analysis of simple patterns. Vision Research. 1986; 26: 1111–1117. 3798746 10.1016/0042-6989(86)90045-3

[50] Theeuwes J., Godijn R. Attentional and oculomotor capture In Folk C. & Gibson B. (Eds.), Attraction, distraction, and action: Multiple perspectives on attentional capture. Amsterdam: Elsevier; 2001; pp.121–149

[51] Yantis S., Jonides J. Abrupt visual onset and selective attention: Evidence from visual search. Journal of Experimental Psychology: Human Perception and Performance. 1984; 10: 601–621. 6238122 10.1037//0096-1523.10.5.601

[52] Wright R.D., Ward L.M. The control of visual attention In Wright R. D. (Ed.), Visual attention. New York: Oxford University Press; 1998; pp. 132–186.

[53] da Rocha E.S., Machado Á.S., Franco P.S., Guadagnin E.C. and Carpes F.P., 2013 Gait asymmetry during dual-task obstacle crossing in the young and elderly. Human Movement, 14(2), pp.138–143.

[54] Nagano H., Begg R.K., Sparrow W.A. and Taylor S., 2011 Ageing and limb dominance effects on foot-ground clearance during treadmill and overground walking. Clinical Biomechanics, 26(9), pp.962–968. doi: 10.1016/j.clinbiomech.2011.05.013 21719169 10.1016/j.clinbiomech.2011.05.013

[55] Perry M.C., Carville S.F., Smith I.C.H., Rutherford O.M. and Newham D.J., 2007 Strength, power output and symmetry of leg muscles: effect of age and history of falling. European journal of applied physiology, 100(5), pp.553–561. doi: 10.1007/s00421-006-0247-0 16847676 10.1007/s00421-006-0247-0

[56] Sadeghi H., Allard P., Prince F. and Labelle H., 2000 Symmetry and limb dominance in able-bodied gait: a review. Gait & posture, 12(1), pp.34–45. 10996295 10.1016/s0966-6362(00)00070-9

